# Case Report of Cystic Mesenteric Lymphangioma as a Cause of Small Bowel Obstruction in an Adult with a Virgin Abdomen

**DOI:** 10.3390/reports9020154

**Published:** 2026-05-18

**Authors:** Niharika Singh, James Petrancosta, Sunjida Ahmed, Nicholas Ahn

**Affiliations:** 1 Department of Surgery, Stony Brook University Hospital, Stony Brook, NY 11794, USA; 2 Department of Pathology, Stony Brook University Hospital, Stony Brook, NY 11794, USA

**Keywords:** small bowel obstruction, cystic mesenteric lymphangioma, surgical emergency

## Abstract

**Background and Clinical Significance:** Cystic mesenteric lymphangiomas are rare benign growths of the mesenteric lymphatic vessels. This entity poses a diagnostic dilemma due to the wide array of symptoms with which patients present. Usually, these patients are diagnosed before the age of 5 years old. **Case Presentation:** In this report, we present a young adult male with longstanding gastrointestinal complaints and a small bowel obstruction who subsequently underwent exploratory laparotomy with significant bowel resection, and pathology revealed a diagnosis of cystic mesenteric lymphangioma. **Conclusions:** The presence of small bowel obstruction without prior abdominal surgeries should raise suspicion of congenital pathologies and warrants prompt surgical intervention.

## 1. Introduction and Clinical Significance

Cystic mesenteric lymphangiomas are congenital defects characterized by the benign proliferation of mesenteric lymphatic vessels and surrounding tissue, causing subsequent lymphatic obstruction and progressive dilation of a multiseptated mass [1,2]. The broader class of cystic lymphangioma is usually noted outside the peritoneum, with the neck, face, axilla, and thoracic regions accounting for 95% of cases. Localization to the mesentery accounts for less than 5% of all lymphatic malformations and under 1% of all lymphangiomas [3,4,5]. The diagnosis of cystic mesenteric lymphangioma most often occurs in children under the age of 5, with 60% of cases established before the first year of life [6,7]. Its incidence is cited as 1 in 100,000 hospital admissions, with a male-female ratio of 3:1 [4,5]. Recent studies have shown that the small bowel mesentery is most commonly involved, with over half of abdominal mesenteric lymphangiomas involving the small intestine [3,4,8].

Common presenting symptoms of cystic mesenteric lymphangioma include abdominal pain and chronic abdominal distension, with a palpable abdominal mass occasionally present [6]. However, given these non-specific presenting symptoms in addition to often atypical clinical manifestations, early diagnosis tends to be challenging [9]. Therefore, many cases are identified either incidentally or upon the emergence of complications such as intestinal obstruction, intracystic infection, hemorrhage, urinary tract obstruction, or volvulus [3,4]. The workup of cystic mesenteric lymphangioma includes radiographic imaging to further characterize the origin and position of each mass, and histological evaluation for a definitive diagnosis and to rule out malignancy. While abdominal CT is widely accepted as the gold standard imaging modality for the diagnosis of cystic mesenteric lymphangioma, abdominal ultrasound can be a more accessible and inexpensive first-line option. Abdominal ultrasound may reveal a cystic appearance with well-defined boundaries and multiple thin or thick septations, while abdominal CT often exhibits a homogenous, hypodense mass with thin non-enhancing partitions. MRI can additionally be considered as an adjunctive tool for determining spatial relationships between the cystic masses and surrounding structures [1,3,9]. Histologically, the diagnosis of cystic mesenteric lymphangioma is established using three criteria: a cystic formation must be present, the septa must be made up of a connective stroma containing lymphoid tissue and striated muscle, and the cyst must be lined with lymphatic endothelial tissue that is positive for the marker D2-40, a marker of lymphatic epithelium [2].

We present a case of a young adult male with longstanding gastrointestinal complaints since childhood presenting with a small bowel obstruction who subsequently underwent an exploratory laparotomy with a 173 cm bowel resection, with pathology revealing a diagnosis of cystic mesenteric lymphangioma.

## 2. Case Presentation

This case is of a 23-year-old white male who presented to the emergency department for abdominal pain that started acutely. The pain had started the night before but continued to increase in severity. He had three episodes of nonbilious non-bloody vomiting overnight. His last bowel movement was the day prior to presentation. He denied night sweats, weight loss, and fevers. Of note, the patient stated he had occasional episodes of severe abdominal pain since he was 11 years old. He had been worked up for gastritis versus cyclic vomiting disorder versus constipation at that time, and was eventually diagnosed with gastritis on esophagogastroduodenoscopy (EGD). He had no other medical problems nor prior abdominal surgeries. He took no medications and had no notable family or social history. On presentation, the patient had a softly distended abdomen with no tenderness. Labs were notable for leukocytosis of 16.6.

**Figure 1:**
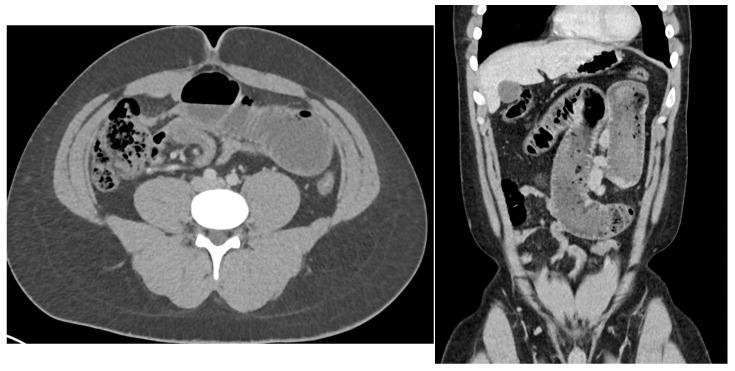
CT abdomen/pelvis in the axial and coronal views revealing a high-grade small bowel obstruction with mesenteric twisting.

**Figure 2:**
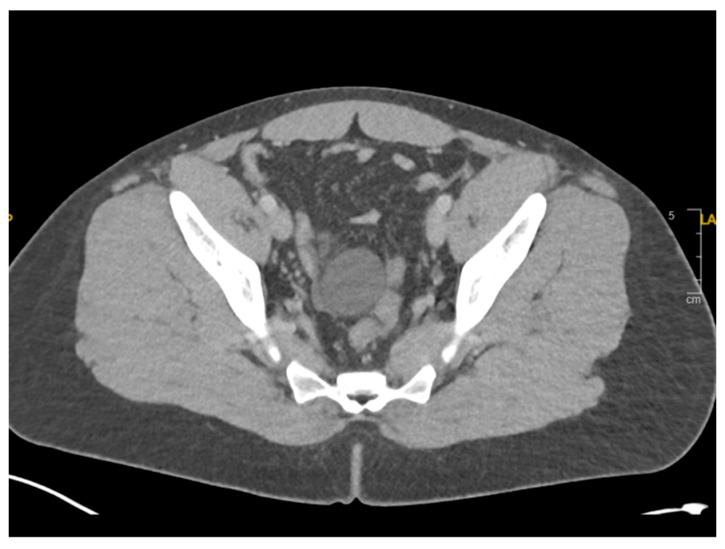
CT abdomen/pelvis in the axial view showed a 4.2 cm cyst in the pelvis, which was not the point of obstruction.

CT Abdomen/Pelvis with intravenous contrast was performed, which showed a high-grade small bowel obstruction with 360-degree rotation of the small bowel at the transition point around its mesentery (Figure 1). There were also a few cystic lesions in the pelvis, the largest of which was 4.2 cm (Figure 2).

**Figure 3:**
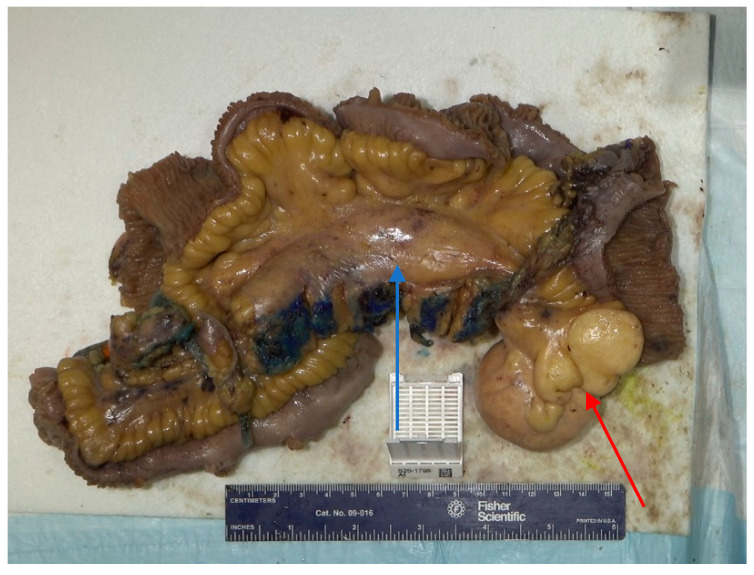
Gross image of the firm mesenteric mass (blue arrow) which corresponds to dilated lymphatics with surrounding adhesions, 173 cm resected bowel, and cystic mesenteric lymphangioma (red arrow) after fixation.

A nasogastric tube was placed with a return of over 1 L of fluid. Due to the high-grade nature of the obstruction and twisting around the mesentery, the patient was taken to the operating room. A diagnostic laparoscopy was performed, which revealed small bowel entering under the mesentery of the distal ileum, suggesting an internal hernia. Due to the degree of dilation, the case was converted to an exploratory laparotomy. The terminal ileum was identified and the bowel was run proximally. At that point, a firm 6 × 8 cm mass in the middle of the ileal mesentery was identified, which was causing nearby scar tissue with internal herniation and mesenteric twisting. Mesenteric windows at the ileum proximal and distal to the segments involved with the mesenteric mass were created. A large multiloculated cyst was included in the resection margin and 173 cm of ileum was resected (Figure 3). An ileal side-to-side anastomosis was created. The remaining bowel was run and it was noted that he had 297 cm of bowel remaining. The fascia and skin were closed.

**Figure 4:**
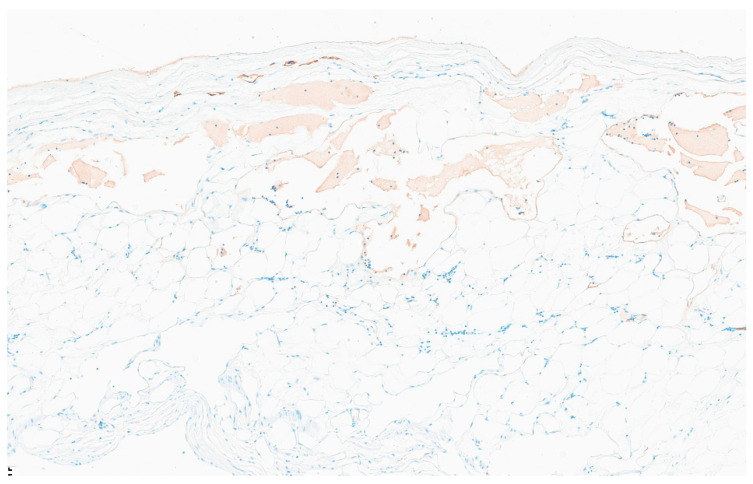
2× D2-40 immunohistochemical stain of the mesenteric cyst supporting lymphatic epithelial lining of the multiloculated cyst, consistent with cystic mesenteric lymphangioma.

**Figure 5:**
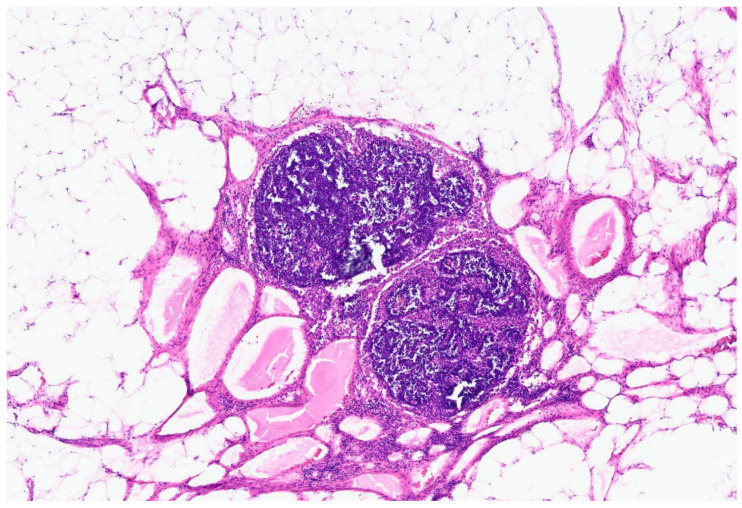
4× H&E image of mesenteric mass revealing multiple lymph nodes with reactive dilated lymphatics.

The patient had an uncomplicated hospital course. On postoperative day 3, the patient had a return of bowel function and the nasogastric tube was removed. By postoperative day 5, the patient was ambulating independently, tolerating foods and was discharged. Two weeks after discharge, the patient was doing well without any repeat vomiting episodes. Pathology was consistent with a 7.0 cm cystic mesenteric lymphangioma with a nearby mesenteric mass with dilated lymphatics. Opening of the cyst revealed a multiloculated cyst with thin, milky white content. Histopathologic examination showed a cyst wall lined by a single layer of flattened endothelium positive for D2-40, supporting a lymphatic epithelial lining (Figure 4). There were also multiple lymph nodes with reactive dilated lymphatic vessels in the firm mesenteric mass (Figure 5).

## 3. Discussion

In this report, we describe an adult male with a history of abdominal pain and vomiting since childhood, who was found to have a 7 cm cystic mesenteric lymphangioma and dilated lymphatics causing a small bowel obstruction. This case is unusual due to the late age of diagnosis. Most cases of cystic mesenteric lymphangioma are diagnosed in children under five years and have a male predominance [4,5,6,7]. Due to the rarity of this disease, the patient had been misdiagnosed with gastritis since he was 11 years old. Potentially, an MRI in the outpatient workup of this patient as an adolescent could have allowed for earlier detection [1,3,9]. Among adults diagnosed with cystic lymphangiomas, Mede et al. found that the mean age was 43 years old; it was more commonly found in females, and only one-third were symptomatic [10]. This patient was significantly younger than other adults diagnosed with this pathology and presented acutely with a high-grade small bowel obstruction.

In our case, the cystic mesenteric lymphangioma was not the direct cause of the obstruction; rather, the firm mass of dilated lymphatics in the root of the mesentery had surrounding adhesions causing an internal hernia. The size and location of mesenteric lymphatic malformations determine the surgical approach and operative methods, with the goal being complete macroscopic excision for large or symptomatic lesions [4,11,12]. Invasion of the lymphangioma into the intestinal wall or infiltration into the mesenteric vessels often requires segmental resection [13]. In our case, due to the involvement of the mesenteric vessels by the dilated lymphatics, a significant portion of the bowel had to be resected (173 cm); however, the patient had sufficient remaining bowel length to prevent short bowel syndrome. Another consideration includes the risk of internal herniation between skeletonized mesenteric vessels.

Recurrence of cystic mesenteric lymphangioma is not uncommon in the postoperative period. Rates of recurrence have been documented at 40% after incomplete resection and 17% after macroscopic complete resection [13]. In adults with cystic lymphangioma, a recurrence rate of 9.4% was observed in the follow-up after surgery [14]. Despite relatively high recurrence rates, there have been no documented cases of malignant transformation [9].

In the setting of high recurrence rates, less invasive treatment modalities are under study in the management of cystic mesenteric lymphangiomas, including ultrasound-guided fine needle aspiration and sclerotherapy [15]. While aspiration can be useful for symptomatic control in the setting of unresectable disease, recurrence rates can be up to 100% [2]. Sclerotherapy is a treatment option involving aspiration of cystic fluid coupled with the injection of a sclerosing agent (bleomycin, doxycycline, or OK-432) into the cystic cavity, inducing degeneration of the endothelial cells in the cyst wall. Recent studies have shown favorable results in reducing the symptoms and size of abdominal lymphangiomas, highlighting its emergence as an alternative therapy for patients with unresectable disease or as an additional therapy to be used in conjunction with surgical resection [16,17].

Macroscopic complete resection was obtained in this case, but due to the risk of recurrence, the patient will continue to follow-up with their gastroenterologist for surveillance. If the mesenteric lymphangioma recurs, sclerotherapy can be utilized to prevent size progression [15,17].

Limitations of this study include the fact that it is a single case with a lack of long-term follow-up. However, this case highlights the presentation, risk factors, surgical options, and post-operative management of the rare pathology of cystic mesenteric lymphangioma.

## 4. Conclusions

We showcase a case of a young adult with longstanding gastrointestinal complaints who presented to the emergency department with a small bowel obstruction, was ultimately taken to the operating room for an exploratory laparotomy and small bowel resection, with pathology confirming cystic mesenteric lymphangioma.

## Data Availability

The original contributions presented in this study are included in the article. Further inquiries can be directed to the corresponding author.
